# The X factor in neurodegeneration

**DOI:** 10.1084/jem.20211488

**Published:** 2022-11-04

**Authors:** Rhonda Voskuhl, Yuichiro Itoh

**Affiliations:** 1 Department of Neurology, David Geffen School of Medicine, University of California, Los Angeles, Los Angeles, CA

## Abstract

Given the aging population, it is important to better understand neurodegeneration in aging healthy people and to address the increasing incidence of neurodegenerative diseases. It is imperative to apply novel strategies to identify neuroprotective therapeutics. The study of sex differences in neurodegeneration can reveal new candidate treatment targets tailored for women and men. Sex chromosome effects on neurodegeneration remain understudied and represent a promising frontier for discovery. Here, we will review sex differences in neurodegeneration, focusing on the study of sex chromosome effects in the context of declining levels of sex hormones during aging.

## Teaser

The study of sex differences in neurodegeneration can reveal treatment targets tailored for women and men. Sex differences in neurodegeneration are reviewed, focusing on sex chromosome effects in the context of declining levels of sex hormones during aging.

## Introduction

The study of sex differences is a way to capitalize on a known clinical observation, mechanistically disentangle it at the laboratory bench, and then translate findings back to the clinic as a novel treatment trial tailored for each sex, a “Bedside to Bench to Bedside” approach (Voskuhl, 2020; Voskuhl and Gold, 2012). The importance of sex as a biologic variable has been recognized by the National Institutes of Health (Clayton, 2016; Clayton and Collins, 2014). Studying sex differences brings scientific rigor and clinical relevance. If a given disease mechanism is discovered not only in one sex but instead in both sexes, then it is relevant to the entire population. On the other hand, if a mechanism is prominent in one sex but not the other, then this is an invaluable clue toward discovery of a potential disease modifier that can be optimized for the relevant sex.

Sex differences occur during health and disease. These sex differences can be mediated by biologic effects, environmental effects, or both. Observations of sex differences across species, for example, between female and male mice in a vivarium underscore the role of biologic effects.

Biologic sex differences can be due to sex chromosomes (XX versus XY), sex hormones (estrogen versus testosterone), or both. Sex chromosomes and sex hormones can act in a synergistic or antagonistic manner on a given process (Palaszynski et al., 2005). Compensatory mechanisms may have arisen during evolution to promote survival of each sex, reaching an optimal balance between sex chromosome and sex hormone influences, which is distinct for each sex (De Vries, 2004). Effects of sex chromosomes and sex hormones are cell-specific and tissue-specific. In diseases that involve multiple organ systems, a deleterious versus beneficial effect of being female or male on disease must be determined in each tissue. For example in multiple sclerosis (MS), an autoimmune disease that attacks the central nervous system (CNS), the effect of a given sex chromosome or sex hormone on disease may differ based on its influence on inflammation in the peripheral immune system versus neurodegeneration in the CNS. Analysis of data from the Genotype-Tissue Expression project examined sex differences in gene expression across 44 tissues in humans and showed that 37% of all genes exhibit sex-biased expression in at least one tissue (Oliva et al., 2020). In another study using the same dataset focusing on 29 human healthy tissues, whole-genome expression profiles showed distinct sex-biased regulatory networks in each tissue (Lopes-Ramos et al., 2020). Furthermore, sex differences in gene expression are region-specific and cell-specific within the brain (Kim-Hellmuth et al., 2020; Oliva et al., 2020). These studies underscore the pervasiveness and complexity of sex differences in gene expression during health with implications for sex differences in neurodegenerative diseases, which can be distinct depending on the brain regions and cells involved. Determining the contribution of sex chromosomes and sex hormones to sex differences in neurodegenerative diseases is a new frontier in the development of novel therapeutics optimally tailored for women and men.

Here, we review sex differences in neurodegeneration and discuss how sex chromosomes modulate autosomal gene expression and phenotype, and then propose future directions to identify brain region–specific and cell-specific mechanisms of neurodegeneration in each sex.

## Sex differences in the brain during health and disease

Sex differences in the brain occur across species, from humans to mice (Corre et al., 2016; Gur et al., 1999; Luders and Toga, 2010; Luders et al., 2014; Meyer et al., 2017; Spring et al., 2007; Voskuhl and Klein, 2019), providing evidence for biological differences due to sex hormones or sex chromosomes. Healthy male brains are on average larger than those of females, maintaining proportion relative to body size. That said, there are also regional differences in substructure volumes even when accounting for differences in brain size. For example, dorsal (posterior) versus ventral (anterior) hippocampus differ regarding which is larger in each sex (Kurth et al., 2017; Meyer et al., 2017; Spring et al., 2007). Sex differences in brain structure have also been found in adolescents (ages 9–10 yr old), as shown by analysis of data from the Adolescent Brain Cognitive Development study (Brennan et al., 2021). The role of sex differences in brain structure during health as it pertains to sex differences during neurodevelopmental disorders and neurodegenerative diseases is an area of active investigation (Wierenga et al., 2022). In addition, sex differences in brain structure during health are critical to take into account when comparing brain substructures during disease. The comparison between substructure volumes in females versus males with disease is confounded by the sex difference during health. To remove this confound, substructure volumes should be compared between females with disease versus females that are healthy as well as between males with disease versus males that are healthy. This permits subsequent determination of whether there is a sex difference in the effect of disease on substructure atrophy (Voskuhl et al., 2020).

Beyond brain structure, there are sex differences at the functional, cellular, and molecular levels. Resting-state functional connectivity using functional magnetic resonance imaging (fMRI) has shown sex differences during health, which may impact the CNS response to a disease. Using imaging data from the Human Connectome Project and the 1000BRAINS study, sex aligned with region-specific differences in brain connectivity. Brain regions most distinct between the sexes included the cingulate cortex, medial and lateral frontal cortex, temporoparietal regions, insula, and precuneus (Weis et al., 2020). In other studies, males displayed more between-module connectivity, while females demonstrated more within-module connectivity, which aligned with sex differences in performance on cognitive domain-specific testing (Satterthwaite et al., 2015). Also, during aging, the default-mode network showed changes in connectivity in both males and females, but at different rates (Scheinost et al., 2015).

Sex differences in brain at the cellular and molecular levels are vast and have been the topic of reviews for decades (Cosgrove et al., 2007; McCarthy et al., 2017). Previous studies focused on sex hormone effects, beginning with localization of sex hormone receptor expression within brain, initially estrogen receptor α (ERα), then ERβ (Merchenthaler et al., 2004; Mitra et al., 2003; Shughrue et al., 2002). Recently it has become apparent that a CNS cell-specific approach to hormone receptor expression in vivo must be determined in each region given the known regional heterogeneity of microglia (Grabert et al., 2016), astrocytes (Chai et al., 2017; Khakh and Sofroniew, 2015), oligodendrocytes (Marques et al., 2016; Vigano et al., 2013), and neurons (Ko et al., 2013). Indeed, sex differences in sex hormone receptor expression should be evaluated similar to sex differences in transcriptomics, namely in a CNS region-specific and cell-specific manner (Kim-Hellmuth et al., 2020; Oliva et al., 2020). This is challenging because sex hormone receptor expression is variable since sex hormones can affect the level of expression of their own receptor. Sex hormone receptor expression can be affected by menstrual cycle phase, menopause, and andropause. Sex hormone receptor expression can also be altered during brain injury, as shown by the upregulation of ERα in astrocytes (Azcoitia et al., 2010; DonCarlos et al., 2006; Garcia-Ovejero et al., 2002). Beyond hormone receptor expression and ligation, function depends on tissue-specific and cell-specific intracellular transcription factors and signaling. For example, ligation of ERα versus ERβ can be synergistic in some tissues and antagonistic in others (Paech et al., 1997; Shang and Brown, 2002; Tiwari-Woodruff et al., 2007). Beyond sex hormones, a new avenue of sex differences research in the brain is a region-specific and cell-specific approach to sex chromosome gene expression.

There are sex differences in brain not only during health but also during neurodegenerative diseases (Voskuhl and Klein, 2019; Young and Pfaff, 2014). In MS, despite the fact that women have more robust peripheral immune responses (Klein and Flanagan, 2016; Libert et al., 2010; Moldovan et al., 2008; Pelfrey et al., 2002) and are more susceptible to disease (Krysko et al., 2020; Voskuhl and Gold, 2012; Whitacre et al., 1999), MS men have worse disability progression (Confavreux et al., 2003; Koch et al., 2010; Ribbons et al., 2015; Weinshenker, 1994). Regarding timing, subcortical gray matter atrophy and cognitive deficits are worse in MS men during young adulthood to midlife (Beatty and Aupperle, 2002; Savettieri et al., 2004; Schoonheim et al., 2012; Voskuhl et al., 2020). In contrast, older MS women have a worsening of their MS disabilities after menopause (Baroncini et al., 2019; Bove et al., 2014a; Bove et al., 2016a; Bove et al., 2016b; Graves et al., 2018; Holmqvist et al., 2006; Smith and Studd, 1992).

Cognitive deficits occur in healthy women with menopause, which have been quantified by performance on objective cognitive tests of verbal memory and processing speed (Dumas et al., 2008; Epperson et al., 2013; Greendale et al., 2009; Greendale et al., 2010; Miller et al., 2013; Rasgon et al., 2005; Wroolie et al., 2011). This is termed “brain fog” and is consistent with loss of neuroprotective estradiol with menopause (Bove et al., 2014b; Halbreich et al., 1995; Miller et al., 2013; Rasgon et al., 2005; Sherwin, 2009; Sherwin et al., 2011; Wroolie et al., 2011).

Alzheimer’s disease (AD) is more common in females, which is not accounted for merely by greater longevity in females (Mielke et al., 2014; Snyder et al., 2016; Uchoa et al., 2016). However, men may be at greater risk for mild cognitive impairment at younger ages (Mielke et al., 2014). Also, at younger ages, the rate of progression from mild cognitive impairment to AD is higher in men, while at older ages the rate is higher in women (Snyder et al., 2016). Loss of endogenous sex hormones in menopausal women and andropausal men is associated with cognitive decline and increased AD risk (Carter et al., 2012; Pike, 2017; Vest and Pike, 2013). Similar deleterious effects of menopause and andropause may be due to testosterone’s conversion to estradiol in brain by aromatase, and thus lower levels of either hormone during aging can decrease ER ligation in brain (Spence and Voskuhl, 2012). This is not mutually exclusive of an effect of lower testosterone levels on ligation of androgen receptors in men (Cherrier et al., 2005).

Parkinson’s disease (PD) has a higher incidence in males than females with a ratio of 2:1 (Oltra et al., 2022; Shulman and Bhat, 2006), and progression of degeneration of the nigrostriatal system is thought to be worse in men (Jurado-Coronel et al., 2018). Men with PD have more severe cognitive impairment, namely executive function and processing speed as measured by the Montreal Cognitive Assessment and the Symbol Digits Modalities Test, respectively (Oltra et al., 2022; Oltra et al., 2021; Reekes et al., 2020). Also, PD males have worse cortical thinning in postcentral and precentral regions and smaller volumes in thalamus, caudate, putamen, pallidum, hippocampus, and brainstem compared with PD females (Oltra et al., 2022; Oltra et al., 2021). Whether testosterone deficiency in aging males or exposure to pesticides that act via estrogen-blocking properties can predispose to PD is a subject of speculation (Brenner, 2012; Okun et al., 2004; Okun et al., 2006; Okun et al., 2002). ER ligands have been proposed as possible candidate future treatments in PD (Bourque et al., 2019; Currie et al., 2004; McFarland et al., 2013).

## Sex differences in the brain with aging

**Figure 1.**
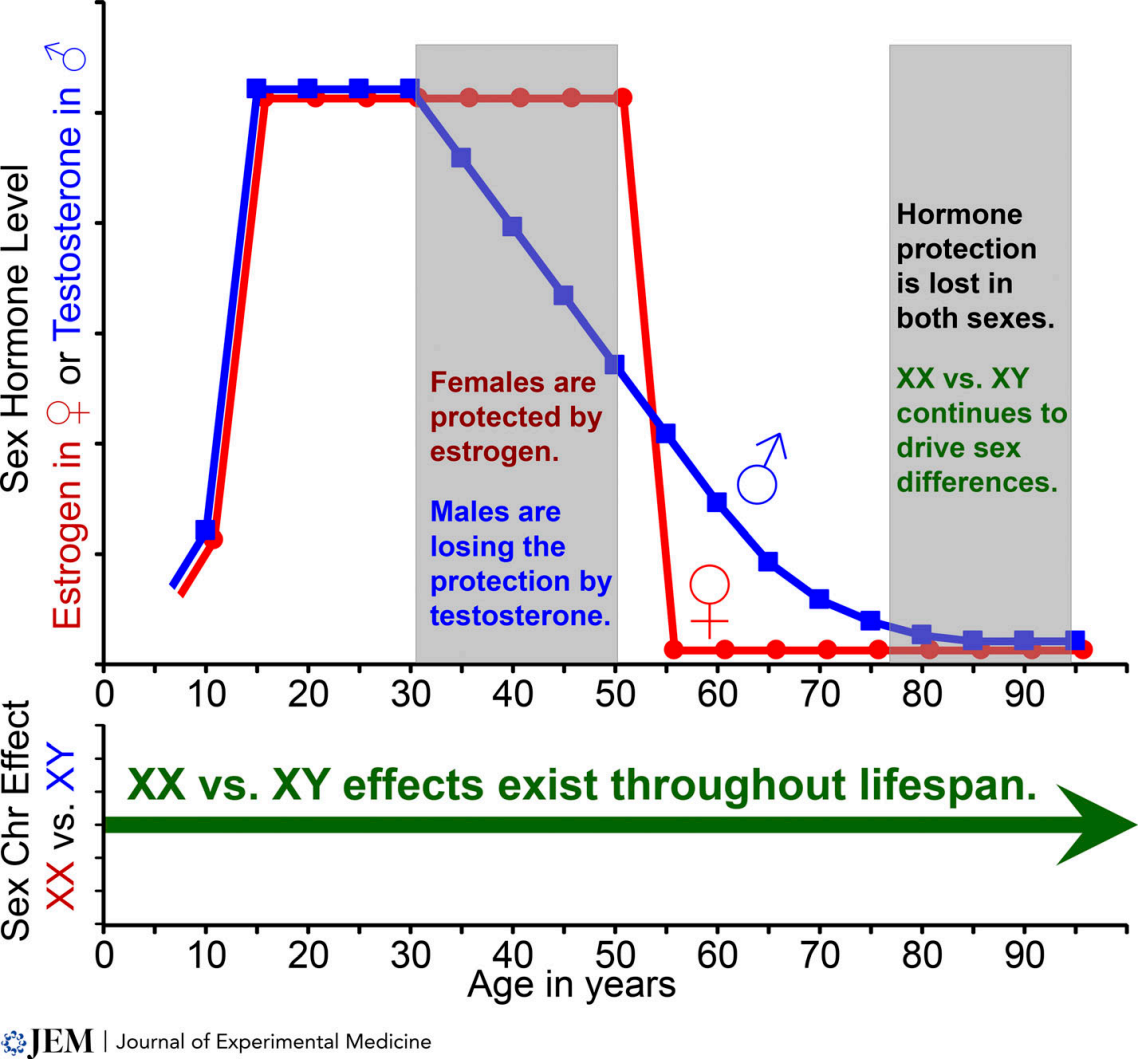
**Sex hormone and sex chromosome effects.** Age-related changes in sex hormones (estrogen versus testosterone) in the context of sex chromosome (XX versus XY) effects across the lifespan. Estrogen, red; testosterone, blue; sex chromosomes, green.

Aging is associated with brain atrophy, neurodegeneration, and cognitive decline in healthy people. It is also the most important risk factor for susceptibility to neurodegenerative diseases. How sex hormones (estrogen, testosterone) and sex chromosomes (XX, XY) influence neurodegeneration across the lifespan remains unclear. We hypothesize that the effect of biological sex across the lifespan is complex, with sex hormones and sex chromosomes contributing differently depending on the timing of the loss of neuroprotective sex hormones during menopause versus andropause. Andropause starts at age 30 with gradual decline of testosterone to old age (Fig. 1, blue). Menopause starts later (ages 46–52) with an abrupt decline in estrogen (Fig. 1, red). In age 75–90 yr, loss of neuroprotective sex hormones in both sexes may unmask underlying effects of sex chromosomes (XX versus XY) that persist across the lifespan (Fig. 1, green). Aging female and male C57BL/6 WT mice underwent in vivo MRI to assess regional brain atrophy and were assessed for underlying neuropathology as well as cognitive impairment. An age-by-sex hormone interaction was discovered (Itoh et al., 2022). Ovariectomized females demonstrated dorsal hippocampal atrophy at midlife, but not young age, which was associated with worse spatial memory on behavioral testing and more glial activation and synaptic loss on neuropathology. Deletion of ERβ in astrocytes, but not neurons, recapitulated these deficits in midlife females. Since sex hormones have been studied more extensively than sex chromosomes, we will now focus on sex chromosome effects on neurodegeneration. That said, observations made when investigating the role of sex chromosomes in neurodegeneration must take into account coexisting effects of sex hormones (Fig. 1).

## The study of sex chromosomes independent of sex hormones

**Figure 2.**
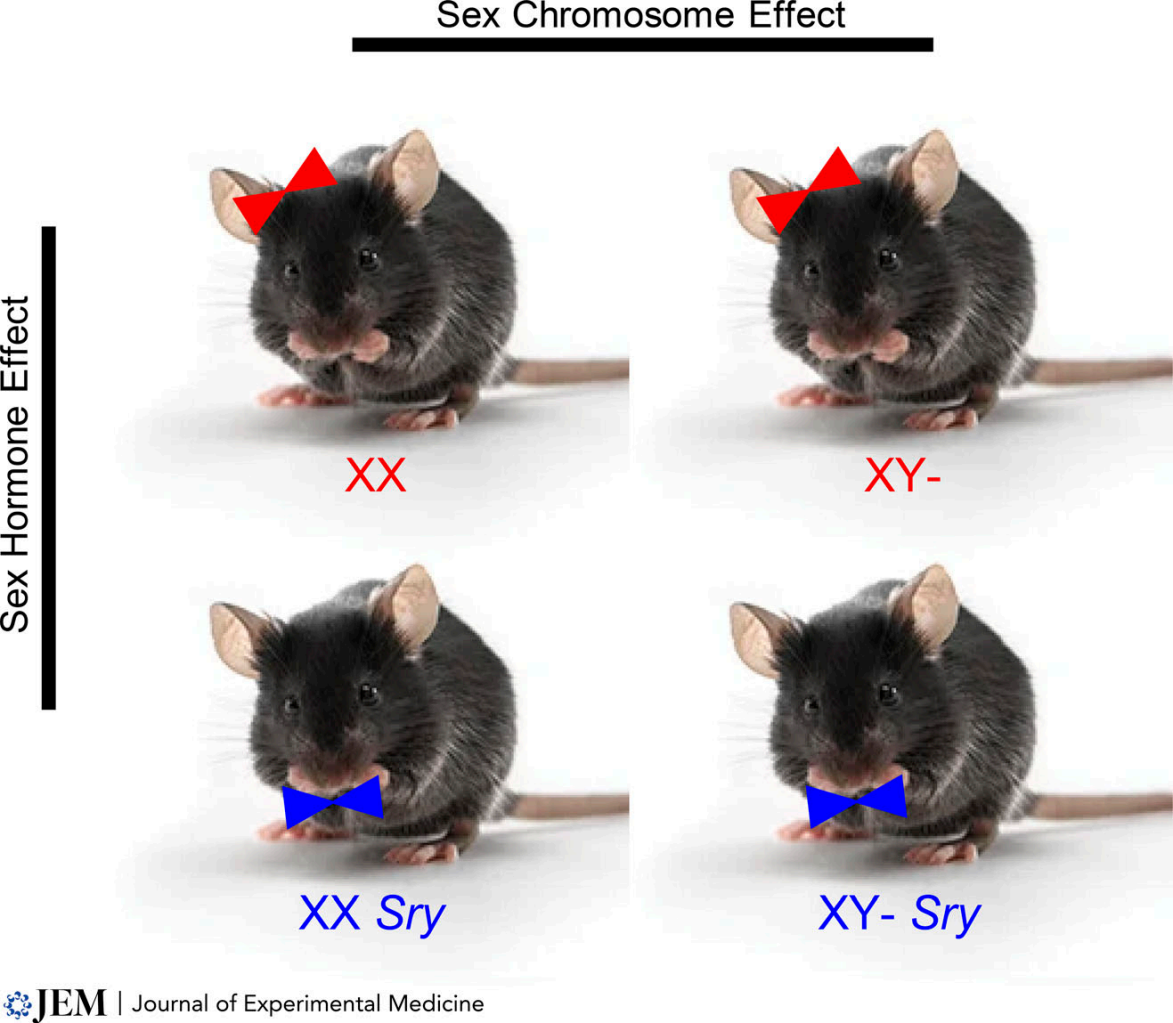
**FCG mice.** The Y chromosome gene that encodes for testicular development (*Sry*) is deleted, with mice designated XY−. Both XX and XY− mice are ovary-bearing (red). Comparisons between XX versus XY− mice sharing a common gonadal type (females throughout life) reveal sex chromosome effects. When the *Sry* transgene is added back at an autosomal location, designated *Sry*, both XX *Sry* and XY− *Sry* mice are testes bearing (blue). Comparisons between XX *Sry* versus XY− *Sry* mice sharing a common gonadal type (males throughout life) also reveal sex chromosome effects. Comparisons between mice with a common sex chromosome complement, with or without the *Sry* gene, reveal sex hormone effects (ovary bearing versus testes bearing throughout life). Comparisons between mice with a common sex chromosome complement that are gonadectomized reveal developmental (organizational) effects of sex hormones (not shown).

Since XX females have ovaries (estrogen) and XY males have testes (testosterone), it is important to remove the confound of a difference in sex hormones when studying sex chromosome effects. Sex hormone effects occur both during development (organizational) and adulthood (activational). Gonadectomy of females and males during adulthood does not control for sex hormone effects during development (prior to gonadectomy). Elegant studies have established how to disentangle the effect of XX versus XY sex chromosome complement from the effect of gonadal type (sex hormones; Smith-Bouvier et al., 2008) using the Four Core Genotype (FCG) mice (Arnold, 2004; Arnold and Burgoyne, 2004). This model has been used worldwide to study sex differences in health (development and adulthood) and diseases (autoimmunity, cardiovascular, and metabolic, to name a few; Blencowe et al., 2022). The Y chromosome gene that encodes for testicular development (*sex determining* *region Y*, *Sry*) is deleted, with mice designated XY−, and they are ovary-bearing (gonadal females). Comparisons can be made between XX versus XY− mice that differ in sex chromosome complement, while sharing a common gonadal type (females throughout life; Fig. 2). If the *Sry* transgene is added back at an autosomal location, comparisons can be made between XX *Sry* versus XY− *Sry* mice that differ in sex chromosome complement, again sharing a common gonadal type (males throughout life; Fig. 2). Comparisons between XX versus XY− mice can be made in gonadally intact mice, as well as in gonadectomized mice, since removal of sex hormones may unmask an effect of sex chromosomes, making an XX versus XY− difference more prominent. Opposing effects of sex hormones and sex chromosomes suggest that even when there is no sex difference in disease in WT, gonadally intact mice, the study of sex hormones and sex chromosomes is still warranted. The two sexes can overall be in balance, yet may differ in the underlying influence of sex hormones and sex chromosomes to reach that balance.

Given the known female bias in several autoimmune diseases (Klein and Flanagan, 2016; Whitacre et al., 1999), sex chromosome effects in peripheral blood immune responses have been studied. Experimental autoimmune encephalomyelitis (EAE) is a prototypic model to study autoantigen-specific immune responses in MS. The relapsing–remitting EAE model on the SJL background was used to examine the role of sex chromosomes on immune responses by backcrossing FCG mice onto the SJL strain. Active EAE was worse in XX mice compared with XY−. Adoptive transfer of autoantigen-stimulated XX lymph node cells, compared to XY− cells, to WT females induced worse clinical disease and neuropathology along with decreased T helper 2 (Th2) anti-inflammatory cytokines, IL 10 and IL 13 (Smith-Bouvier et al., 2008). These studies demonstrated a role for sex chromosomes in the immune induction phase of adoptive EAE, with the XX complement, compared to XY−, more proinflammatory. A proinflammatory effect of XX compared to XY− was also shown in experimental (Smith-Bouvier et al., 2008) and spontaneous (Sasidhar et al., 2012) lupus models using the FCG model.

Sex differences vary depending on autosomal genetic background (or strain of mouse). In contrast to SJL mice, where females show a more severe clinical course, there is no sex difference in EAE walking scores in C57BL/6 mice (Palaszynski et al., 2004; Papenfuss et al., 2004). Yet, even in C57BL/6 mice, androgens exert anti-inflammatory effects on cytokines and peroxisome proliferator-activated receptor α (PPARα) in T lymphocytes in the immune system (Doroshenko et al., 2021; Dunn et al., 2007; Zhang et al., 2012). Conversely, XY sex chromosome complement in the CNS confers a worse neurodegenerative response to immune-mediated injury (Du et al., 2014). If a male sex hormone and the male sex chromosome complement exert opposing effects on disease in a given strain (De Vries, 2005; Palaszynski et al., 2005), then there may not be a sex difference when comparing males to females. This demonstrates why disease mechanisms in each sex should be studied even in the absence of an overall sex difference in disease.

## A deep dive into XX versus XY mediated sex differences

Differences between XX versus XY sex chromosome complements may be due to (1) the presence or absence of the Y chromosome, (2) differential imprinting of X genes based on maternal (X_m_) versus paternal (X_p_) parent-of-origin in X_m_X_p_ versus X_m_Y, and (3) X dosage effects. Much focus is on the X chromosome instead of Y since the Y chromosome has evolved to lose most of its genes, except for those involved in male reproduction (Burgoyne, 1998). In contrast, the X chromosome contains about 10% of the human genome including a rich repository of genes, many with immune functions (*TLR7* and *TLR8*; *cluster differentiation 40 ligand*; *Forkhead box P3*; *C-X-C Motif Chemokine Receptor 3*; Jiwrajka and Anguera, 2022) and others that are highly expressed in brain (*Synaptophysin*; *Synapsin*; *Synapse Associated Protein 1*; *Proteolipid Protein 1*; *Monoamine Oxidase A and B*; Collenberg et al., 2019; Fassio et al., 2011; Mallajosyula et al., 2008; Nguyen and Disteche, 2006a; Nguyen and Disteche, 2006b; Sun et al., 2020; Tarsa and Goda, 2002; Tatar et al., 2010). Regarding differential imprinting of X genes based on parent-of-origin (maternal versus paternal), transcriptomes of stimulated CD4^+^ T lymphocytes showed higher expression of a cluster of X genes when derived from XY as compared with XX mice, opposite the direction of an X-dosage effect. An increase in DNA methylation spanning many regions of the X chromosome of paternal origin (X_p_), as compared with maternal origin (X_m_), was found (Golden et al., 2019). DNA methylation usually suppresses gene expression, so this result was consistent with higher expression of a cluster of X genes in X_m_Y cells because all cells from X_m_Y mice express from the X_m_, so have minimal suppression. In contrast, X_m_X_p_ mice have half of their cells expressing from the X_m_ and half from the X_p_ due to random X chromosome inactivation, so the overall cell population has relatively more suppression. The implications of these findings are that parent-of-origin differences in DNA methylation of the X chromosome can lead to sex differences in X gene expression, namely higher expression in X_m_Y than X_m_X_p_ for a cluster of genes (Golden et al., 2019; Voskuhl et al., 2018).

The effect of having two X chromosomes on susceptibility to systemic lupus erythematosus, which has a female to male sex bias of ∼9:1, has been reviewed (Jiwrajka and Anguera, 2022). A role for partial inactivation of immune genes on the X chromosome resulted in a partial dosage effect of TLR7, with higher expression in XX than XY (Souyris et al., 2018; Souyris et al., 2019). Also, skewed X chromosome inactivation has been observed in immune cells of females with rheumatologic diseases, whereby there is preferential (non-random, not 50%) activation of the maternal versus the paternal X chromosome (70% or more; Amos-Landgraf et al., 2006). Implications for skewed X-inactivation on gene expression at the cell population level are magnified by the potential for parent-of-origin differences in methylation and its effect on expression of X chromosome genes (Golden et al., 2019; Jiwrajka and Anguera, 2022; Voskuhl et al., 2018).

X-escapees are X chromosome genes known to escape X-inactivation (3% in mice and 15% in humans; Berletch et al., 2011). Sex differences in health and disease could be due to the dosage of known X-escapees, with higher expression in XX than XY. Lysine demethylase 6A (*Kdm6a*) is an X-chromosome gene known to escape X-inactivation (Disteche et al., 2002; Greenfield et al., 1998). This gene encodes for a histone demethylase that removes suppressive histone marks to broadly upregulate both autosomal and sex chromosome gene expression. Selective deletion of *Kdm6a* in CD4^+^ T lymphocytes in active EAE in C57BL/6 mice reduced walking disability and decreased neuropathology in spinal cord. RNA sequencing of CD4^+^ T lymphocytes from conditional knockout (CKO) versus WT revealed the downregulation of expression of neuroinflammation signaling pathway genes (*Purinergic Receptor*; *C-X-C Motif Chemokine Ligand 10*; *Tlr1*, *Amyloid Precursor Protein* [*APP*]). Peripheral immune responses in the CKO showed less memory and more naive phenotype, including lower expression of CD44 on CD62L^+^ T cells. Analysis of chromatin immunoprecipitation sequencing data showed more repressive histone H3 lysine trimethylation (H3K27me3) modifications on the CD44 gene in the CKO (Itoh et al., 2019). Thus, *Kdm6a* was shown to be proinflammatory in CD4^+^ T lymphocytes of C57BL/6 mice and to confer worse neurodegeneration in the chronic EAE model. Interestingly, in a different EAE model, when transgenic Th17 T lymphocytes were adoptively transferred into non-obese diabetic.*Scid* mice, Th17 cells from males induced more severe disease, and there was a higher frequency of pathogenic, IFNγ producing Th17 cells as compared with those from females. Use of gonadectomy and the FCG model revealed that XY− genotype, not exposure to androgens, was responsible for the generation of more encephalitogenic Th17 cells and worse EAE severity in this model (Doss et al., 2021). *Kdm5c*, an X-escapee that is a histone H3 lysine 4 demethylase (Berletch et al., 2015), was then overexpressed in male Th17 cells using a retroviral vector, and this reduced Th17 pathogenicity and EAE severity. Thus, higher doses of *Kdm5c* were protective in transgenic Th17 cells (Doss et al., 2021). Another report showed that by restricting H3K4me3 modification at core promoters, *Kdm5c* dampens transcription, but at enhancers, *Kdm5c* stimulates their activity in mouse embryonic stem cells and neuronal progenitor cells (Outchkourov et al., 2013). Together, these findings reveal how X-escapees can regulate gene expression to impact immune responses and neurodegeneration.

## Sex chromosome effects in the CNS

A significantly higher proportion of genes on the X chromosome, as compared to genes on autosomes, are preferentially expressed in the brain compared to other somatic tissues (Nguyen and Disteche, 2006a; Nguyen and Disteche, 2006b). The accumulation of brain-specific genes located on the X chromosome over evolution puts them in a unique position to influence the CNS response to injury. In EAE or MS, that injury would be an immune attack. The CNS response to an immune attack involves microglial and astrocyte activation, demyelination, axonal damage, and synaptic loss in MS (Chomyk et al., 2017; Dutta et al., 2011; Mori et al., 2013; Trapp et al., 2007) and EAE (Centonze et al., 2010; Meyer et al., 2020; Nistico et al., 2013; Rasmussen et al., 2007; Ziehn et al., 2012a; Ziehn et al., 2012b; Ziehn et al., 2010). A sex chromosome complement effect in the CNS during EAE was shown using the FCG model (Du et al., 2014). Bone marrow chimeras were created to study sex chromosome effects in the CNS, not confounded by differences in the immune system. Specifically, XX versus XY− bone marrow chimeras were reconstituted with a common immune system of one sex chromosome complement. EAE mice with XY− sex chromosome complement in the CNS, compared with XX, demonstrated worse EAE clinical severity with more neuropathology in spinal cord (axonal and myelin loss), cerebellum (Purkinje cell and myelin loss), and cerebral cortex (synaptic loss). This was the first demonstration of an effect of sex chromosome complement on neurodegeneration in a neurodegenerative disease. These data coincide with clinical observations in humans that while females (XX) are more susceptible to MS, men (XY) have worse disability progression (Confavreux et al., 2003; Koch et al., 2010; Ribbons et al., 2015; Voskuhl et al., 2020; Weinshenker, 1994).

In an AD model where mice express human APP (hAPP), the FCG model showed that mice of the XY− sex chromosome complement had worse mortality and cognitive deficits than XX (Davis et al., 2020). Also, lentivirus vector-induced knockdown of *Kdm6a* expression in XX neurons worsened amyloid β (Aβ)–mediated neuronal toxicity using in vitro assays, while *Kdm6a* overexpression in XY− neurons reduced toxicity. This was consistent with a dose effect whereby two copies of *Kdm6a* in XX were protective as compared with one copy in XY−. Also, when *Kdm6a* was overexpressed through lentivirus injection into the dentate gyrus in vivo, there were less cognitive deficits in XY−hAPP mice. The CNS cell that overexpressed *Kdm6a* after injection into the dentate gyrus was not determined. Further study is warranted using a cell-specific knockout of physiologic levels of *Kdm6a* in neurons in vivo in XX-hAPP mice.

Together, the above reveals a deleterious effect of the XY sex chromosome complement in the CNS in MS and AD models, but the CNS cell type mediating this effect in vivo remains unclear. Further, since selective deletion of the X-escapee *Kdm6a* in CD4^+^ T lymphocytes in C57BL/6 mice ameliorated EAE, revealing a deleterious effect of *Kdm6a* on immune-mediated neurodegeneration, it is important to do selective deletion of the *Kdm6a* in each CNS cell to ascertain the effect of *Kdm6a* on neurodegeneration in a tissue-specific and cell-specific manner in each disease. Since *Kdm6a* can regulate autosomal gene expression, this is consistent with studies showing sex differences in gene expression which are tissue-specific and cell-specific during health (Kim-Hellmuth et al., 2020; Oliva et al., 2020).

## Sex differences in microglia

Microglia, the resident immune cells of the CNS, lie at the intersection of immune and neurodegenerative mechanisms. Microglia can confer beneficial and deleterious effects during normal development and disease (Hammond et al., 2019; Hong et al., 2016; Schafer et al., 2013; Shi et al., 2017; Stevens and Schafer, 2018; Vasek et al., 2016). Microglia become activated in white and gray matter to play a critical role in neurodegenerative conditions (Absinta et al., 2021; Hui et al., 2020; Jackle et al., 2020; Priller and Prinz, 2019; Schirmer et al., 2021; Yanguas-Casas et al., 2020). Deleterious effects of microglia in MS and EAE include creating a proinflammatory environment that inhibits oligodendrocytes from remyelinating axons in white matter lesions (Absinta et al., 2021; Kim et al., 2018). Brain MRI has identified paramagnetic phase rims lesions in white matter of MS patients, thought to reflect ongoing microglial damage that contributes to worsening disability (de Haan and Karnath, 2018; Ruggieri et al., 2018). Specifically, lesions with outer rims indicate iron accumulation in microglia and macrophages, thereby serving as a biomarker for these lesions having chronic-active inflammation. A sex difference was reported whereby white matter lesions visualized in men were significantly more likely to have paramagnetic phase rims than lesions in women (Tolaymat et al., 2020). Deleterious effects of microglia activation in gray matter include synaptic engulfment and synaptic loss (Schirmer et al., 2021; Werneburg et al., 2020). On the other hand, beneficial effects of microglia include clearance of myelin debris and other molecules toxic to neurons and oligodendrocytes (Dong and Yong, 2019; Rawji et al., 2020).

Microglial activation is also an important component of brain aging and AD (Grabert et al., 2016; Mangold et al., 2017; Mishra and Brinton, 2018; Pan et al., 2020). Microglia are thought to play a beneficial role in early stages of AD and a deleterious role in later stages (Keren-Shaul et al., 2017; Stephen et al., 2019). Genetic risks for sporadic, late-onset AD have been linked to Triggering Receptor Expressed on Myeloid Cells 2 (TREM2), which is expressed in microglia (Jin et al., 2014; Jonsson et al., 2013; Nguyen et al., 2020; Prokop et al., 2019; Ulrich et al., 2017). Extracellular Aβ deposition is one of the earliest pathologies and precedes cognitive decline. Activation of TREM2 in microglia limits Aβ-mediated damage through plaque compaction and clearance (Shi and Holtzman, 2018). Another genetic risk factor is inheritance of allele 4 of apolipoprotein E (APOE4; Krasemann et al., 2017). The APOE4 isoform is associated with higher Aβ levels in brain than APOE3 (Stephen et al., 2019). Higher levels of Aβ aggregates are thought to stimulate microglia activation which could have deleterious effects on inducing oxidative stress, mitochondrial damage, and synaptic loss (Shi and Holtzman, 2018). A sex by APOE genotype interaction has also been described. Namely, the beneficial effect of microglial on plaque compaction in an AD mouse model was significantly less in female mice with the APOE4 genotype (Stephen et al., 2019). This aligns with clinical data that women who are APOE4 carriers have increased risk for AD (Riedel et al., 2016). The role of microglia in the progression of AD as it relates to APOE genotype, sex, and aging remains unclear and is of major interest, as reviewed (Chen et al., 2021; Patel et al., 2022). Other sex differences in microglia have also been reviewed in aging and AD (Delage et al., 2021; Lynch, 2022; Yanguas-Casas et al., 2020). The regions of focus are hippocampus and prefrontal cortex in the context of effects on cognition. A variety of sex differences have been described, most frequently involving the level and functional type of microglial activation and its association with synaptic phagocytosis and loss.

Estrogens and androgens have been shown to reduce microglial activation (Christensen et al., 2020; Drew and Chavis, 2000; Kim et al., 2018; Wu et al., 2013; Ziehn et al., 2012b), but less is known about sex chromosome effects in microglia. Expression of *Kdm6a/KDM6A* in microglia in mice and humans was examined by our group using an existing RNA sequencing dataset for the microglia transcriptome from forebrain (GSE117646) of RiboTag mice (Kang et al., 2018). The mean *Kdm6a* expression level in microglia was 32.07 transcripts per million (TPM) in females and 23.04 TPM in males (P = 4.27 × 10^−12^), whereas the average TPM for all expressed genes was 8.18 TPM in females and 8.18 TPM in males. In humans, using the existing RNA sequencing dataset for microglia isolated from corpus callosum (GSE111972), the mean expression of *KDM6A* gene was 8.52 TPM in females and 6.19 TPM in males (P = 0.000857), while the mean TPM for all expressed genes was 3.14 TPM in females and 3.19 TPM in males. Together, this demonstrated that *Kdm6a* and *KDM6A* are expressed in microglia in mice and humans, respectively, and indeed showed a dosage effect with higher expression in females than males. Selective deletion of *Kdm6a* in microglia is now warranted to determine its effect on aging and models of neurodegenerative diseases.

## Concluding remarks and future directions

A cell-specific, region-specific, and sex-specific approach to neurodegeneration is warranted and is consistent with the sex bias in gene expression observed in humans during health (Kim-Hellmuth et al., 2020; Oliva et al., 2020). There are gene expression differences from one brain region to another in neurons (Ko et al., 2013), microglia (Grabert et al., 2016), astrocytes (Chai et al., 2017; Khakh and Sofroniew, 2015), and oligodendrocytes (Marques et al., 2016; Vigano et al., 2013). A difference in gene expression in astrocytes from one brain region to another during neurodegenerative disease was first shown in EAE (Itoh et al., 2018). Spinal cord astrocytes had decreased expression of cholesterol synthesis genes, and treatments targeting a cholesterol transporter improved clinical scores and decreased neuropathology (Itoh et al., 2018). RNA sequencing of astrocytes from optic nerve identified the complement pathway as a potential target, and sex differences suggested that treatments targeting the complement pathway during optic neuritis may be more effective in females (Tassoni et al., 2019). RNA sequencing of oligodendrocytes in corpus callosum during remyelination after cuprizone-mediated demyelination suggested targeting estrogen response elements of cholesterol synthesis genes as a strategy for enhancing remyelination (Voskuhl et al., 2019). These examples provide evidence that understanding the effect of biological sex on cell-specific and region-specific transcriptomes in the CNS can point to novel treatments targeting neurodegeneration optimized for women and men.

**Figure 3.**
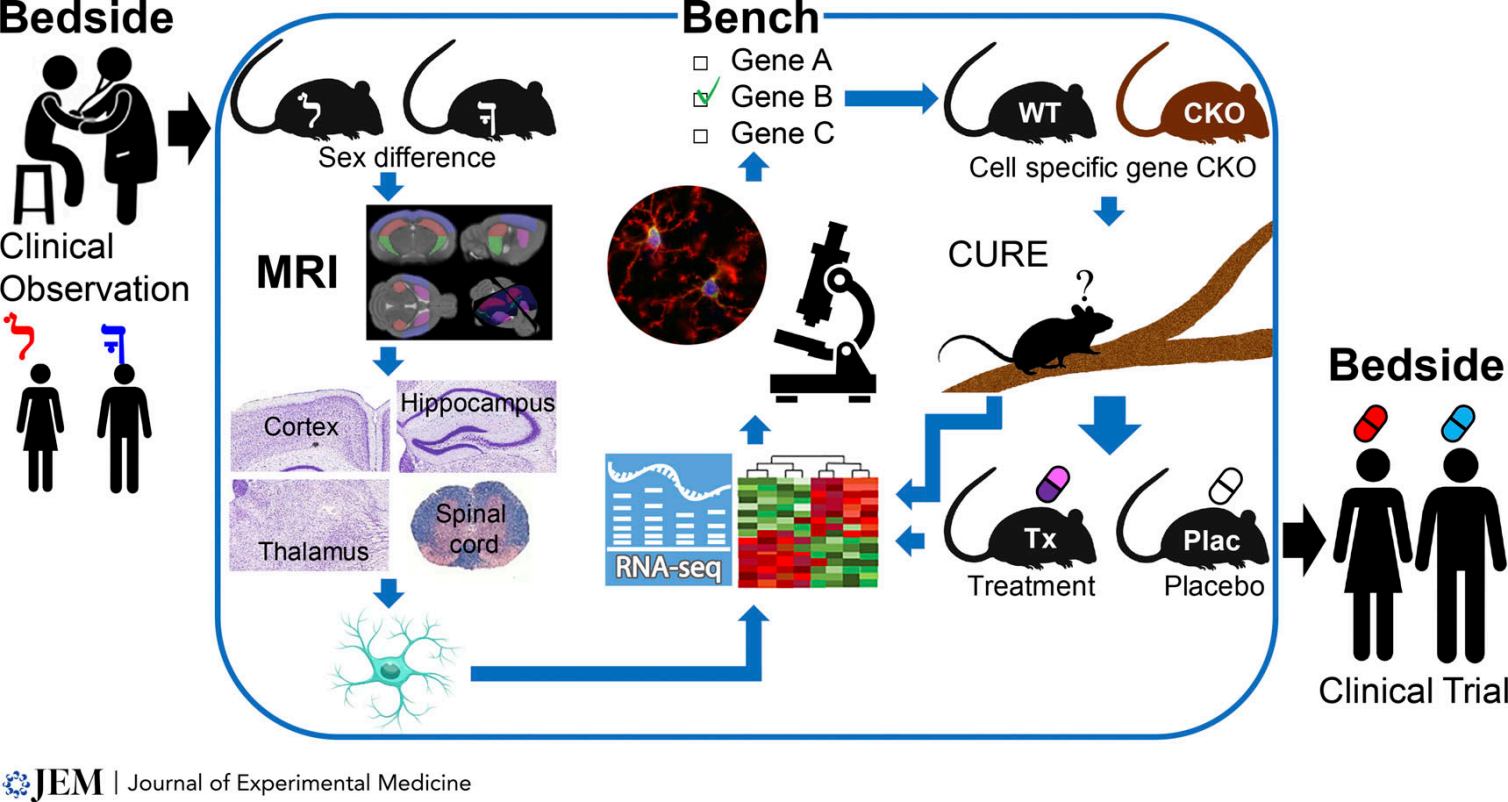
**Bedside to Bench to Bedside to study sex differences in neurodegeneration: Region-specific, cell-specific, and sex-specific research.** Clinical observations of sex differences are investigated at the preclinical level and then translated back to the clinic as trials designed for each sex. Bench investigations entail in vivo MRI for region-specific atrophy, neuropathology of each region, RNA sequencing of each CNS cell from each region, immunohistochemistry validation of genes in top differentially expressed pathways, knockout of target gene in each CNS cell (CKO) to reverse phenotype, and knockdown of target gene with pharmacologic treatment (Tx) to reverse phenotype. Reiteration can determine the effect of genetic (CKO versus WT) and/or pharmacologic (treatment versus placebo) intervention on reversal of gene expression using the same cell-specific and region-specific approach in each sex. Also, replacement of female versus male mice in the beginning with gonadectomized versus gonadally intact mice will reveal activational effects of sex hormones, while FCG mice will reveal developmental hormone effects or sex chromosome effects, each in a region-specific and cell-specific manner.

Since microglia reside at the intersection of immune and neurodegenerative mechanisms, they are a critical cell in the study of sex differences in neurodegeneration. Future directions warrant a region-specific and sex-specific approach to the study of microglia in health versus disease, as shown in Fig. 3. Such preclinical findings can serve as a basis for translation to clinical trials in humans. This will inform whether sex hormones, sex chromosomes, or both contribute to sex differences in neurodegeneration across the lifespan, as shown in Fig. 1, paving the way for discovery of precision treatments tailored to achieve neuroprotection in women and men.
